# Which chronic diseases and disease combinations are specific to multimorbidity in the elderly? Results of a claims data based cross-sectional study in Germany

**DOI:** 10.1186/1471-2458-11-101

**Published:** 2011-02-14

**Authors:** Hendrik van den Bussche, Daniela Koller, Tina Kolonko, Heike Hansen, Karl Wegscheider, Gerd Glaeske, Eike-Christin von Leitner, Ingmar Schäfer, Gerhard Schön

**Affiliations:** 1 Department of Primary Medical Care, Center of Psychosocial Medicine, University Medical Center Hamburg-Eppendorf, Martinistrasse 52, 20246 Hamburg, Germany; 2 Division of Health Economics, Health Policy and Outcomes Research, Centre for Social Policy Research, University of Bremen, Parkallee 39, 28209 Bremen, Germany; 3 Department of Medical Biometry and Epidemiology, Center for Experimental Medicine, University Medical Center Hamburg-Eppendorf, Martinistrasse 52, 20246 Hamburg, Germany

## Abstract

**Background:**

Growing interest in multimorbidity is observable in industrialized countries. For Germany, the increasing attention still goes still hand in hand with a small number of studies on multimorbidity. The authors report the first results of a cross-sectional study on a large sample of policy holders (n = 123,224) of a statutory health insurance company operating nationwide. This is the first comprehensive study addressing multimorbidity on the basis of German claims data. The main research question was to find out which chronic diseases and disease combinations are specific to multimorbidity in the elderly.

**Methods:**

The study is based on the claims data of all insured policy holders aged 65 and older (n = 123,224). Adjustment for age and gender was performed for the German population in 2004. A person was defined as multimorbid if she/he had at least 3 diagnoses out of a list of 46 chronic conditions in three or more quarters within the one-year observation period. Prevalences and risk-ratios were calculated for the multimorbid and non-multimorbid samples in order to identify diagnoses more specific to multimorbidity and to detect excess prevalences of multimorbidity patterns.

**Results:**

62% of the sample was multimorbid. Women in general and patients receiving statutory nursing care due to disability are overrepresented in the multimorbid sample. Out of the possible 15,180 combinations of three chronic conditions, 15,024 (99%) were found in the database. Regardless of this wide variety of combinations, the most prevalent individual chronic conditions do also dominate the combinations: Triads of the six most prevalent individual chronic conditions (hypertension, lipid metabolism disorders, chronic low back pain, diabetes mellitus, osteoarthritis and chronic ischemic heart disease) span the disease spectrum of 42% of the multimorbid sample. Gender differences were minor. Observed-to-expected ratios were highest when purine/pyrimidine metabolism disorders/gout and osteoarthritis were part of the multimorbidity patterns.

**Conclusions:**

The above list of dominating chronic conditions and their combinations could present a pragmatic start for the development of needed guidelines related to multimorbidity.

## Background

Driven by increasing longevity and the rise of healthcare costs, a growing interest in multimorbidity is observable in industrialized countries [1]. Still, there is a far smaller number of studies on multimorbidity than on individual chronic diseases [2]. Pioneering work on multimorbidity has been done in a few countries, in particular Australia [3,4], Canada [5,6], The Netherlands [7,8], and Sweden [9,10]. All studies demonstrate a high prevalence of chronic diseases especially among the elderly. However, they differ largely with regard to the figures on prevalence and the number of chronic diseases. For example, in a literature review published in 2005, Fortin et al. found rates of multimorbidity in the elderly of 49% to 99% and an average number of chronic diseases per person between 2.5 and 6.5 [5]. Most studies also show a strong association between the degree of multimorbidity and its impact on patients, e.g. on mortality, functional status and quality of life [6,11,12]. The broad range of prevalences in studies is due to differences in study design and the chronic conditions included. For example, mental comorbidity is often not investigated, although it is frequently associated with somatic morbidity [12-14].

This growing interest in multimorbidity contrasts with the small number of studies in Germany too. Since 2008, the Federal Ministry of Education and Research has been supporting six research networks studying health in the elderly, in which multimorbidity is an important topic [15]. This paper reports the concept and the first results of a study on multimorbidity in the elderly (65 years and older) using routine claims data from a German statutory health insurance company. The aim of the study was to explore the field of multimorbidity for Germany and to develop strategies for further investigations, as this was the first systematic study on multimorbidity in the elderly in Germany.

## Methods

The basis for the analysis are the claims data of the Gmünder ErsatzKasse (GEK), a statutory health insurance company operating nationwide in Germany and insuring some 1.7 million persons, a figure corresponding to some 2.4% of the statutorily insured population. The data were provided by GEK-Insurance in a pseudonymous form.

Originally, the GEK insured mainly craftsmen, and therefore the proportion of insured men exceeds that of women even today. Previous studies have shown that results from the GEK database can be transferred to the German population as a whole, if adjusted for age and gender [16]. For this study, adjustment was performed for age and gender for the general German population according to data from the Federal Bureau of Statistics as of December 31, 2004. The adjustment procedure and its results are shown in Additional File 1.

The claims data stem from physicians in the ambulatory care setting only. Claims are forwarded on a quarterly basis by the individual physician to the association of statutory health insurance physicians, where they are checked for comprehensiveness and plausibility. Thereafter, the data are transmitted to the statutory insurance companies. According to German law, physicians in the ambulatory care setting [they are not receiving care] must note all ICD-10 codes relevant to the current treatment of the individual patient in the electronic claim document. Since elderly multimorbid patients visit physician premises in ambulatory care with an average frequency of 36 contacts per year, the registration of morbidity by physicians in this study is most likely not impeded by a lack of contacts.

We selected the most frequent conditions in GP surgeries as published in a panel survey ("ADT Panel") of the Central Research Institute of Statutory Ambulatory Health Care in Germany [17,18]. Chronicity of diagnoses was assessed using the "Expert Report for the Selection of 50 to 80 Diseases to be Included in the Morbidity Based Risk Adjustment Scheme", published in 2007 [19]. In order to capture a comprehensive picture of the disease patterns in individual patients we amended this list for all chronic conditions with a prevalence ≥ 1% in the age group ≥ 65 years found in the GEK data set of from 2006. The ICD-10 codes for chronic conditions were grouped by an expert panel of family physicians from the Hamburg Institute of Primary Medical Care in order to account for coding variance among physicians for the same syndrome. For example, F00-F03, F05.1, G30, G31 and R54 were grouped under the heading "dementia". The result of this procedure was a list of 46 single codes and code groups further referred to as "chronic conditions" in this paper. This list includes all frequent somatic and psychic disorders (see Additional File 2).

A person was defined as chronically ill if she/he had at least one of the 46 chronic conditions in at least three quarters within the one-year observation period of 2004. The three-quarters criterion was chosen in order to avoid transitory or erroneous diagnoses, a usual procedure when using health insurance claims data for research in Germany. Also, acute or subacute forms of certain conditions were excluded by using this criterion. Among the chronically ill, a person was considered to be multimorbid when he/she had three or more chronic conditions from the list. The criterion of three chronic conditions was considered to be a more valid cut-off score for multimorbidity in elderly patients treated in the ambulatory care setting, instead of the usual criterion of two chronic conditions [20], which would frequently lead to very high rates of multimorbidity in the age group over 65 years. Recent research supports using the criterion of ≥ 3 conditions for investigations of multimorbidity in the ambulatory care setting [8,21], especially when the aim is to compare multimorbid and non-multimorbid samples in the total cohort.

In our study, adjusted prevalences were calculated separately for the total cohort as well as for the multimorbid sample (abbreviated as mm-sample) and the non-multimorbid sample (abbreviated as nmm-sample). Furthermore, relative risks (or risk ratios; abbreviated RR) were calculated for individual chronic conditions. The relative risk expresses the likelihood of a chronic condition to be associated with multimorbidity (under consideration of the prevalence of the condition) or - in other words - the likelihood of multimorbid persons suffering from a specific chronic condition in comparison to non-multimorbid persons. Due to somewhat high prevalence of several chronic conditions, we preferred the estimation of relative risks over odds ratios [22]. Observed-to-expected ratios (O/E ratio) were calculated in order to estimate conditional probabilities of combinations of three chronic conditions. All analyses were performed using the statistics packages SAS (version 9.2) and R (version 2.11.1). The study was approved by the Ethics Committee of the Medical Association of Hamburg.

## Results

The cohort consisted of 123,224 patients aged 65 years and older. Sociodemographic characteristics of the total cohort and of both the mm-sample and the nmm-sample after adjustment for the German population in 2004 are presented in Table 1.

**Table 1:** Sociodemographic characteristics of the total cohort, the multimorbid and non-multimorbid samples (adjusted for the German population 2004)

	Total cohort	Multimorbid sample	Non-multimorbid sample	p*
Number of persons	123,224	76,540	46,684	
%	100.0%	62.1%	37.9%	

Mean Age all (SD)	74.1 (7.1)	75.1 (7.2)	72.5 (6.7)	< 0.001
Mean age men (SD)	72.7 (6.4)	73.7 (6.5)	71.3 (5.9)	< 0.001
Mean age women (SD)	75.1 (7.5)	76.0 (7.5)	73.4 (7.2)	< 0.001

Gender (% females)	59.2%	61.5%	55.3%	< 0.001

Average number of chronic conditions (SD)	3.9 (3.2)	5.8 (2.6)	0.8 (0.9)	< 0.001
Mean number men (SD)	3.6 (3.1)	5.7 (2.5)	0.7 (0.8)	< 0.001
Mean number women (SD)	4.1 (3.2)	5.9 (2.6)	0.8 (0.9)	< 0.001

Nursing care dependency all (% receiving services)	19.1%	23.2%	12.3%	
% males	14.2%	18.0%	8.8%	< 0.001
% females	22.4%	26.5%	15.1%	< 0.001

SD = standard deviation;

p* = statistical significance of the difference between the multimorbid and the non-multimorbid sample (t-tests were performed for comparison of means, and chi-square-tests for differences between percentages).

Persons in the mm-sample were on average 2.6 years older than those in the nmm-sample. Women were overrepresented among the multimorbid (61.5% vs. 55.3% in the nmm-sample) and were older than men in both samples (2.3 years older in the mm- and 2.1 years in the nmm-sample). The percentage of patients receiving services from the statutory nursing insurance scheme due to disability was almost 100% higher among the multimorbid than among the non-multimorbid.

**Figure 1:**
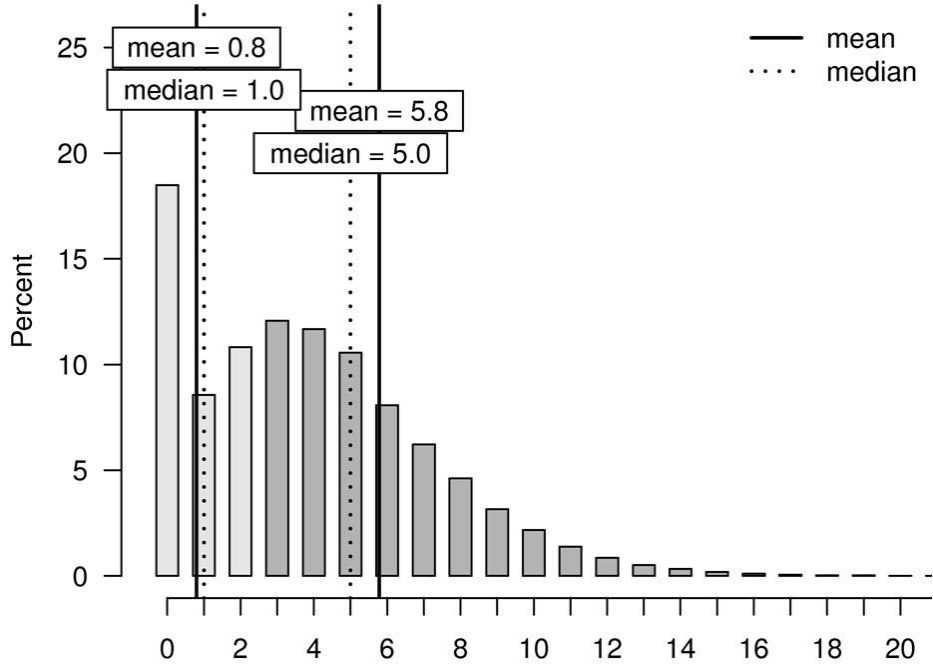
**Frequency (in %) of the number of chronic conditions within the list of 46 conditions in the non-multimorbid sample (light grey columns) and the multimorbid sample (dark grey columns) and mean and median for both samples**.

62.1% of the cohort was multimorbid in the sense of presenting ≥ 3 chronic conditions from the 46-item list (see Figure 1). With a criterion of ≥ 2 chronic conditions this percentage would have risen to 73%. Even with a criterion of ≥ 4 chronic conditions, nearly half (49%) of the sample would have been defined as multimorbid.

The median of chronic conditions in the mm-sample was 5, compared to 1 in the nmm-sample. Gender differences with regard to the number of chronic conditions in the mm-sample (5.7 for males and 5.9 for females) were small but statistically significant (p < 0.001). In the nmm-sample, nearly half (48.8%) had no chronic condition of the list whereas the other half had 1 (22.6%) or 2 (28.6%).

**Figure 2:**
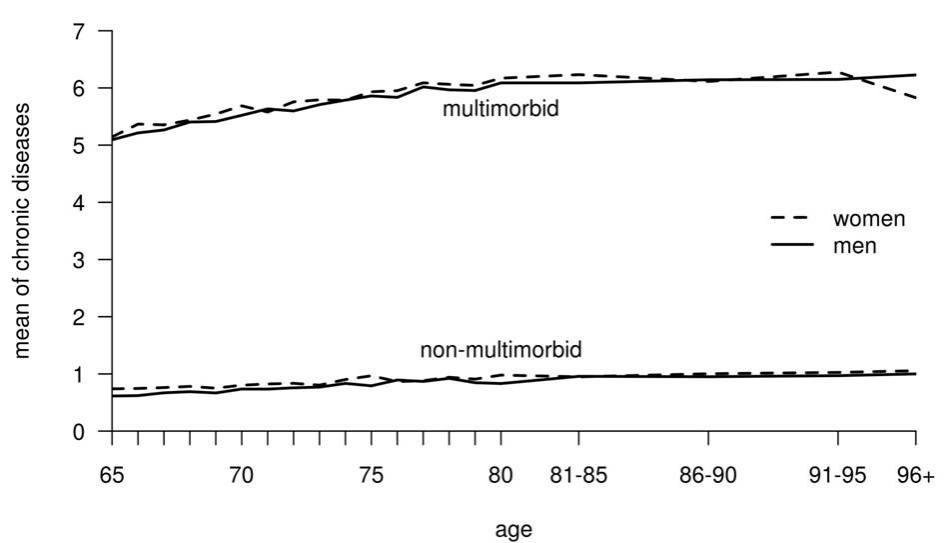
**Mean number of chronic conditions according to age and gender in the non-multimorbid and the multimorbid sample**. Means were calculated for each year of life until 80. Due to the small number of cases in old age, we generated four age groups for age > 80 years before calculation.

As expected, the number of chronic conditions increased with age in the mm-sample (Figure 2). However, the difference in the number of chronic conditions between the youngest and the oldest age-group was only 1, although the difference in average age between the youngest and the oldest age group was 17 years.

### Prevalence of individual chronic conditions

Hypertension was by far the most frequent chronic condition in both samples (65.4% in the mm-sample and 17.9% in the nmm-sample). Lipid metabolism disorders (42.9%) and chronic low back pain (41.2%) were also very frequent among multimorbid patients. A prevalence slightly below 30% was found for osteoarthritis (29.5%), diabetes mellitus (28.5%), and chronic ischemic heart disease (27.5%), whereas severe vision reduction (21.5%) and thyroid dysfunction (21.4%) had a prevalence slightly over 20% in the mm-sample. In total, 21 of the 46 chronic conditions had a prevalence of > 10% in the mm-sample. Adjusted prevalence, prevalence rank order, and relative risk for multimorbidity of the 46 chronic conditions in the multimorbid and non-multimorbid sample are presented in Additional File 3 (for the total cohort), Additional File 4 (for the female subsample) and Additional File 5 (for the male subsample).

### Prevalence of triadic combinations

Combinations of the 46 chronic conditions under study ranged mainly between 3 and 15 in the mm-sample (see Figure 1). Further analysis of multimorbidity was based on combinations of three chronic conditions. In theory, 15,180 such triadic combinations are possible, of which we found 15,024 (99%) in the dataset. Even for combinations of five conditions we found 832,589 of the theoretically possible 1,370,754 (60.7%). Table 2 shows the 10 most prevalent triadic combinations (see Additional File 6 for full data on the 50 most frequent combinations). The most frequent individual chronic conditions also dominate the triadic combinations. The highest prevalence was found for the combination hypertension, lipid metabolism disorders and chronic low back pain (12.1% in the mm-sample and 7.5% in the total cohort). A closer look at the 10 most frequent combinations shows that these three conditions, together with diabetes mellitus, osteoarthritis, and chronic ischemic heart disease, are the main components of the 10 most prevalent triads: hypertension was present in 9 out of 10, lipid metabolism in 6, and chronic low back pain in 5 out of the top ten prevalent triads. Triads of the six most prevalent individual chronic conditions (hypertension, lipid metabolism disorders, chronic low back pain, diabetes mellitus, osteoarthritis and chronic ischemic heart disease) spanned the disease spectrum of 42% of the multimorbid sample. Gender differences were minor: All combinations with osteoarthritis were more prevalent in females, whereas all combinations with chronic ischemic heart disease were more prevalent in men. Also, the combination of hypertension, lipid metabolism disorders and purine/pyrimidine metabolism disorders/gout was the fourth most frequent triad in multimorbid males (prevalence 9.7% in the male mm-sample), whereas this combination ranked 21st among the most frequent combinations in women (prevalence 5.9% in the female mm-sample).

**Table 2:** Adjusted prevalence, prevalence rank order and O/E ratio of the 10 most prevalent triadic combinations of chronic conditions

Combinations of chronic conditions	Rank (1)	Prevalence in total cohort (2)	Prevalence in mm-sample (3)	Prevalence in male mm-sample (4)	Prevalence in female mm-sample (5)	O/E ratio of mm (6)
Hypertension + lipid metabolism disorders + chronic low back pain	1	7.5	12.1	11.1	12.6	1

Hypertension + chronic low back pain + osteoarthritis	2	6.4	10.4	8.1	11.8	1.3

Hypertension + lipid metabolism disorders + chronic ischemic heart disease	3	5.8	9.4	11.9	7.8	1.2

Hypertension + lipid metabolism disorders + diabetes mellitus	4	5.8	9.3	10.2	8.7	1,2

Hypertension + lipid metabolism disorders + osteoarthritis	5	5.4	8.6	6.6	9.9	1

Lipid metabolism disorders + chronic low back pain + osteoarthritis	6	4.7	7.5	6.1	8.4	1.4

Hypertension + lipid metabolism disorders + purine/pyrimidine metabolism disorders/gout	7	4.6	7.3	9.7	-	1.6

Hypertension + chronic low back pain + chronic ischemic heart disease	8	4.5	7.3	8.1	6.8	1

Hypertension + chronic low back pain + diabetes mellitus	9	4.5	7.3	7.1	7.4	1

Hypertension + diabetes mellitus + chronic ischemic heart disease	10	4.3	6.9	8.3	6.0	1,3

For adjustment of the prevalences see Additional File 1; rank order according to prevalence in the sample;

mm = multimorbidity

mm-sample = multimorbid sample; O/E ratio = observed-to-expected ratio

The dominance of a small number of chronic conditions in the combinations is also illustrated by the fact that hypertension alone is present in two-thirds of the 100 most prevalent triads.

### Relative risk for multimorbidity

The relative risk (or risk ratio; RR) expresses the likelihood that a chronic condition will be associated with multimorbidity in consideration of the prevalence of that condition. Table 3 lists the chronic conditions with the highest (cut-off: RR > 15) and the lowest relative risk (RR < 5) for multimorbidity in the mm-sample according to gender and age groups.

**Table 3:** Chronic conditions with high and with low relative risk for multimorbidity according to gender and age groups

Diagnosis group	RR all	RR women	RR men	RR age 65-74	RR age 75+
High relative risk for multimorbidity					

Renal insufficiency	25.5	34.3	23.2	32.6	17.2

Obesity	20.3	19.0	22.1	20.8	26.3

Liver disease	18.1	19.3	17.9	18.4	21.8

Chronic cholecystitis/gallstones	17.8	16.5	19.1	19.5	14.4

Intestinal diverticulosis	17.1	18.8	14.9	16.1	20.0

Urinary tract calculi	16.7	27.0	15.5	17.8	17.9

Purine/pyrimidine metabolism disorders/Gout	16.2	31.4	12.9	15.5	18.2

Atherosclerosis/PAOD	16.1	16.8	16.2	18.0	12.1

Anemia	15.8	17.3	14.5	20.8	10.5

Neuropathy	15.2	14.9	15.6	17.5	11.6

Low relative risk for multimorbidity					

Hypertension	3.7	3.4	4.1	3.9	3.2

Dementias	3.9	3.4	4.9	4.6	2.6

Cancers	4.3	4.4	4.5	4.2	4.2

Severe vision reduction	4.5	4.4	4.6	4.5	4.0

RR = risk ratio (or relative risk)

The risk ratio for multimorbidity was highest for renal insufficiency (25.5) and obesity (20.3). A risk ratio of 25 means that people with this syndrome are 25 times more frequently multimorbid than non-multimorbid. Most chronic conditions had a comparable relative risk in both genders, whereas some had a much higher one among women (purine/pyrimidine disorders, urinary tract calculi and renal insufficiency) (see Additional Files 3, 4, 5 for full data on gender differences). Renal insufficiency, anaemia and obesity had a risk ratio ≥ 20 among those aged under 75 years, whereas obesity, liver disease and intestinal diverticulosis had such a risk ratio in the age group ≥ 75 years. The risk ratio was lowest for hypertension (3.7), dementia (3.9), cancer (4.3) and severe vision reduction (4.5). The risk ratio of dementia was lower for those aged 75 and older compared to those aged 65-74, and also much lower for women than for men.

### Observed-to-expected ratio of frequent combinations

We extracted those triadic combinations with an O/E ratio ≥ 1.5 from the list of the 100 most prevalent combinations and found 16 combinations (see Additional File 6). Such a cutoff score means that the prevalence of a combination was some 50% higher than expected based on the prevalence of the individual conditions. Remarkably, purine/pyrimidine metabolism disorders/gout was present in 7 of the 16 combinations with high excess prevalence, and osteoarthritis was present in 6 of them. It should also be noticed that the majority of triads with a higher O/E ratio were combinations with a relatively low prevalence, as only 3 triads in Additional File 6 belong to the top third of the 100 most prevalent combinations while all others have a relatively low prevalence.

This phenomenon is also illustrated by the fact that only one of the top ten prevalent combinations has a relatively high O/E ratio: 1.6 for the combination hypertension + lipid metabolism disorders + urine/pyrimidine metabolism disorders/gout (see Additional File 7).

## Discussion

The statutorily insured elderly German population examined in this study showed a high degree of multimorbidity, as 62% of the cohort had 3 chronic conditions or more from a list of 46. This high prevalence was found in spite of a cut-off score for multimorbidity that is more selective than in most other studies, which define multimorbidity as a coexistence of ≥ 2 chronic conditions [23]. The median of chronic conditions among the multimorbid was 5, with almost no gender-related and only small age-related differences. Our results are in line with those of the few German studies on multimorbidity in the elderly population. The "Berliner Altersstudie" (Berlin Aging Study - BASE), a representative cohort study on aging in Berlin aged 70 to 95+ that was based on extensive clinical examinations and interviews, found an average prevalence of ≥ 5 chronic conditions from a list of 28 in 88% of their sample [24]. In 2006 the ADT Panel Study of the German Central Institute of Statutory Ambulatory Medical Care found an average of 6 diagnoses in the age group between 60 and 80 visiting a GP [18,19]. Furthermore, the CONTENT Study, in which morbidity was continuously recorded in general practices in 2006 using the International Classification of Primary Care (ICPC), found combinations of hypertension, lipid metabolism disorders, diabetes mellitus, low back pain/osteoarthritis and chronic ischemic heart disease to be the most frequent individual conditions in both genders, also including depression in females [25]. This result matches perfectly with our findings regarding the constituents of the most frequent triadic combinations. The results of the study on multimorbidity published in 2003 by the Robert Koch Institute - the national epidemiological institute financed by the federal government - are hardly comparable since that study was based mainly on patient survey data and was limited to the population aged up to 79 years [26]. Our results are also in line with those from studies in other countries, the majority of which also reported prevalences of multimorbidity between 50% and nearly 100% in the elderly. For example, in 2005 Britt et al. found a 75% prevalence of multimorbidity in the general practice setting, on the basis of 2 chronic conditions, in a sample of Australians aged 65-74 years, and an 83% prevalence for those aged 80 years and more [4]. In a similar setting in Quebec, Fortin et al. found a multimorbidity prevalence on the same basis in 99% of women and in 97% of men for the age-group 65 and older, and of 98% and 91% respectively when using the criterion of 3 conditions [5]. According to Anderson und Horvath, two-thirds of the US population aged 65 years and older suffer from at least two chronic conditions [27], whereas Wolff et al. found that 65% of a Medicare sample had 2 or more, 43% 3 or more, and 24% 4 or more chronic conditions [28].

The marked differences in prevalence figures are due to several kinds of differences in study design. They are related to the population under investigation (e.g. population-based versus GP-patient populations) [8] or the frequency of visits to physicians, particularly specialists. Differences with regard to the definition and inclusion of morbidity may even be more important. The syndromes under investigation are usually defined in a closed list of (chronic) diseases, but the number of included syndromes varies between a figure less than 10 and more than 100 [8,11,20,23]. In some cases, open lists are used. The items included are ICD codes (single or grouped), involved morbidity domains (organs and systems) within multimorbidity indexes, or causes for contact with the GP based on the ICPC [25]. An overview of methods available for the measurement of comorbidity and the assessment of their validity and reliability is provided by de Groot et al. [29]. Also, in many studies the analysis is restricted to numbers, leaving the medical impact and the subjective burden out of scope of investigation, in spite of their relevance from the patient's perspective [30]. Other studies include information on severity and impact on patients [4]. An important difference is also due to the chosen cut-off score for multimorbidity. As described earlier, the presence of ≥ 2 chronic conditions is usually used as a criterion, whereas some use ≥ 3 conditions, especially for GP-populations [8,21]. In a recent study comparing two data sources, Fortin et al. found a prevalence of multimorbidity around 70% in the age group 65-79 years and around 90% in the age group ≥ 80 years in a GP-based sample when using the criterion of ≥ 2 chronic conditions. Using the criterion of ≥ 3 chronic conditions, prevalences were much lower (around 45% in the age group 65-79 years and around 65% in the age group ≥ 80 years). Fortin et al. also found a prevalence increase of around 30% in the age-group 65-79 years when using an open list of diseases instead of a closed list of seven diseases (all figures not exactly reported in the paper but drawn from the figures) [21]. Generally speaking, the more chronic conditions included, the higher the prevalence of multimorbidity should be, whereas a higher cut-off score for multimorbidity (e.g. 3 or 4 instead of 2 chronic conditions) should lead to lower prevalences. Last but not least, different methods for the analysis of the co-occurrence of conditions are used [9]. Because of these multiple differences in approach, research would indeed benefit from a process of agreeing upon the methodology and inclusion criteria of forthcoming studies on multimorbidity [8,21].

The small gender differences with regard to the number of chronic conditions found in this study are in line with the results of Britt et al. [4] and van den Akker et al. [7]. In other studies, however, larger gender differences were found, usually showing a higher prevalence in women [10], but occasionally also in men [5,21]. However, important gender differences were found in this study for individual chronic conditions and combinations: all combinations with osteoarthritis were more prevalent in women, whereas all combinations with chronic ischemic heart disease were more prevalent in men. Also, the prevalence of the combination of hypertension, lipid metabolism disorders and purine/pyrimidine metabolism disorders/gout was much higher in men than in women.

Beyond counting chronic conditions, this study attempted to look more closely at the individual diseases and the development and consequences of (triadic) disease combinations, their possible interactions and synergies. On the one hand, we identified a small number of chronic conditions which were involved in the vast majority of frequent triadic combinations (hypertension, lipid metabolism disorders, chronic low back pain, diabetes mellitus, osteoarthritis, and chronic ischemic heart disease). On the other hand, we found that 99% of the possible combinations of three chronic conditions were indeed present in the data. This means that multimorbidity appears in an almost infinite number of variants with a mostly low prevalence [8], which makes it difficult to draw generalizable conclusions about the medical care needs of multimorbid persons. We identified high-risk ratios for multimorbidity for a certain number of chronic conditions, either in both genders (e.g. obesity and renal insufficiency) or more pronounced in women (e.g. purine/pyrimidine metabolism disorders/gout and urinary tract calculi; see Additional File 3, 4 and 5). In our view it is an important result that, in addition to renal insufficiency, obesity has a high relative risk for multimorbidity in men and women and throughout all age groups. This hits upon the need for prevention and support of patients undertaking lifestyle changes to start long before the age of 65.

The O/E ratio allowed for the detection of excess prevalence not explicable by the mere addition of individual prevalences. In this respect, purine/pyrimidine metabolism disorders/gout and osteoarthritis in particular showed excess prevalence in several combinations. Both conditions should be examined in depth as potential predictors of multimorbidity in further analyses.

On the other hand, some chronic conditions have a low degree of association with multimorbidity. In the case of hypertension, this is due to the fact that hypertension is also frequent in the non-multimorbid population. As for dementia, this condition does not appear in any of the 100 most frequent triadic combinations. The relative "stand-alone" character of dementia in this study is in line with the finding by Marengoni et al. in the Swedish Kungsholmen study, in which dementia had the highest percentage of cases without comorbidity [9]. As stated by Marengoni, this may be due to an underreporting of complaints, such as pain symptoms, by patients with dementia in clinical examinations. However, a long-standing relationship with a GP might lead to another result, as we found that patients with dementia had a significantly higher number of comorbid conditions than controls in German claims data [31].

Our rationale for the investigation of the multimorbidity patterns was formulated by Gijsen et al. in their review on the causes and consequences of comorbidity in 2001: "All studies [...] found a significant effect of comorbidity on mortality, functional status, (and) quality of life. Here [...], it remains largely unclear to which extent generalizable influences of multimorbidity or specific effects of individual disease combinations are responsible for which effects" [12]. We believe that the clinical relevance of future studies on multimorbidity will depend largely on the investigation of the aetiology, as well as the course and the impact of specific combinations of chronic conditions [8,21,32].

This study has a number of limitations but also strengths. We examined a larger spectrum of chronic diseases than most studies. Also, by examining triadic combinations, we approached a more complex spectrum within multimorbidity. However, as this study is based on claims data, the diagnoses were not clinically verified by specially trained professionals. Whereas accidental and erroneous diagnoses could be minimized by excluding codes appearing in less than three quarters of the year, it cannot be excluded that physicians differ with regard to coding quality and thresholds used to qualify a phenomenon as a disease. We assume that clear-cut somatic conditions suffer less from underreporting than, for example, conditions associated with lifestyle, such as obesity or tobacco abuse, or psychic dysfunction [8]. In an exploratory study comparing German claims data with the charts of the GPs in 2003, Erler et al. found an underreporting in 30% of cases [33]. However, underreporting was related mainly to minor medical problems, whereas the degree of correspondence for the "classical" chronic conditions requiring treatment (e.g. diabetes, ischemic heart disease) was high. Privately insured patients (some 10% of the population) were not included in this study. It should also be noted that the prevalence estimates for multimorbidity in this paper are biased by underreporting due to the fact that a person can have other chronic conditions than the 46 on our list. This assumption is supported by the preliminary findings of our MultiCare prospective multicentre cohort study based on physician chart reviews and interviews using an open list. Here, in general, higher prevalences of multimorbidity were detected [24].

On the other hand, our insurance claims data allow analyses of large populations over time, including those living in nursing homes and thus also frail individuals as well as the oldest of the elderly, who are all frequently excluded in field studies. The same applies to the lack of selection bias with regard to the service providers, an even greater problem in field research. Furthermore, recall bias and social desirability problems in surveys and interviews are excluded. Also, the morbidity figures in our study are not biased by differences in the frequency of health services utilization, as all persons included visited a physician during at least three quarters of the year [16]. We also used a relatively long list of chronic conditions, permitting us to include all chronic conditions with a ≥ 1% prevalence, and thus minimizing the bias effect caused by including only high prevalence conditions [20]. To summarize, this is a descriptive, cross-sectional study with limited possibilities of attribution of causalities in the field of multimorbidity. We intend to validate our results by using a longitudinal approach in the analysis of the claims data and by comparing the results with those from the MultiCare prospective multicentre cohort study, which is based on chart reviews and structured interviews with GPs and patients [34].

## Conclusion

There is widespread agreement in the literature that research on multimorbidity should aid in developing specific guidelines for those suffering from multiple chronic conditions. Triads of the six most prevalent individual chronic conditions (hypertension, lipid metabolism disorders, chronic low back pain, diabetes mellitus, osteoarthritis, and chronic ischemic heart disease) correspond to the multimorbidity spectrum of almost half of the multimorbid sample. We propose to use this result as a pragmatic starting point for guideline development in multimorbidity.

## Competing interests

GG received funding from statutory health insurance companies for scientific studies, among them from the GEK. The other authors declare no competing interests.

## Authors' contributions

HvdB, IS, HH and GS conceived and designed the study. GG acquired the data. DK, TK, KW and GS performed the statistical analyses. HvdB drafted the manuscript, all other authors revised it critically and approved the final manuscript.

## Pre-publication history

The pre-publication history for this paper can be accessed here:

http://www.biomedcentral.com/1471-2458/11/101/prepub

## Supplementary Material

Additional file 1 **Data adjustment procedure and results**. Click here for file

Additional file 2 **List of the 46 chronic conditions and their ICD-codes used in this study**. Click here for file

Additional file 3 **Adjusted prevalence, prevalence rank order and relative risk for multimorbidity of the 46 chronic conditions in the multimorbid and non-multimorbid sample ordered according to prevalence in total cohort**. Click here for file

Additional file 4 **Adjusted prevalences, prevalence rank order and relative risk for multimorbidity of the 46 chronic conditions in the multimorbid and non-multimorbid sample in women according to prevalence in women sample**. Click here for file

Additional file 5 **Adjusted prevalences, prevalence rank order and relative risk for multimorbidity of the 46 chronic conditions in the multimorbid and non-multimorbid sample in men according to prevalence in the male sample**. Click here for file

Additional file 6 **Triadic combinations with O/E ratio ≥ 1.5 out of the list of 100 most prevalent combinations in the multimorbid sample**. Click here for file

Additional file 7 **Adjusted prevalence in total cohort and in mm-sample, and O/E-ratio of the 50 most prevalent triadic combinations of chronic conditions out of the list of 46**. Click here for file
