# Supplementary material for: Which chronic diseases and disease combinations are specific to multimorbidity in the elderly? Results of a claims data based cross-sectional study in Germany
Source: BMC Public Health. 2011 Feb 14;11:101. doi: 10.1186/1471-2458-11-101 (PMC3050745; doi:10.1186/1471-2458-11-101)
Supplement: Additional file 1 — Data adjustment procedure and results. [file 1471-2458-11-101-S1.PDF]

**Additional file 1: Data adjustment procedure and results**

To adjust for the age and gender specifics of the GEK database, adjustment was performed for the German general population according to data from the Federal Bureau of Statistics as of December 31, 2004. All analyses were conducted with sampling weights to correct the differences of age and gender. Sampling weights were obtained from the German population data as of December 12, 2004 (http://www.destatis.de). Each year of age for women and men was assigned its own sampling weight, except for ages 85 and older, which share a common weighting factor for age. The following table shows the differences between the unadjusted and adjusted data.

**Table: Comparison of raw and adjusted sociodemographic data**

| | **Raw data** | | | **Adjusted data** | | |
|---|---|---|---|---|---|---|
| | **total cohort** | **mm-sample** | **nmm-sample** | **total cohort** | **mm-sample** | **nmm-sample** |
| Distribution of MM/NMM | 100.0% | 58.9% | 41.1% | 100.0% | 62.1% | 37.9% |
| Percentage of women | 42.4% | 44.6% | 39.2% | 59.2% | 61.5% | 55.3% |
| Age (mean, SD) | 72.0 (6.1) | 72.9 (6.3) | 70.7 (5.6) | 74.1 (7.1) | 75.1 (7.2) | 72.5 (6.7) |
| Female | 72.8 (6.7) | 73.6 (6.8) | 71.4 (6.2) | 75.1 (7.5) | 76.0 (7.5) | 73.4 (7.2) |
| Male | 71.4 (5.7) | 72.3 (5.9) | 70.2 (5.1) | 72.7 (6.4) | 73.7 (6.5) | 71.3 (5.9) |
| Number of chronic diagnoses (mean, SD) | 3.6 (3.1) | 5.7 (2.5) | 0.8 (0.8) | 3.9 (3.2) | 5.8 (2.6) | 0.8 (0.9) |
| Female | 3.9 (3.2) | 5.7 (2.6) | 0.8 (0.9) | 4.1 (3.2) | 5.9 (2.6) | 0.8 (0.9) |
| Male | 3.5 (3.1) | 5.6 (2.5) | 0.7 (0.8) | 3.6 (3.1) | 5.7 (2.5) | 0.7 (0.8) |
| Nursing care dependency | 13.4% | 16.9% | 8.3% | 19.1% | 23.2% | 12.3% |
| Female | 16.0% | 19.6% | 10.3% | 22.4% | 26.5% | 15.1% |
| Male | 11.4% | 14.8% | 7.0% | 14.2% | 18.0% | 8.8% |

mm-sample = multimorbid sample; nmm-sample = non-multimorbid sample; SD = standard deviation
