# Supplementary material for: Which chronic diseases and disease combinations are specific to multimorbidity in the elderly? Results of a claims data based cross-sectional study in Germany
Source: BMC Public Health. 2011 Feb 14;11:101. doi: 10.1186/1471-2458-11-101 (PMC3050745; doi:10.1186/1471-2458-11-101)
Supplement: Additional file 3 — Adjusted prevalence, prevalence rank order and relative risk for multimorbidity of the 46 chronic conditions in the multimorbid and non-multimorbid sample ordered according to prevalence in total cohort. [file 1471-2458-11-101-S3.PDF]

Additional File 3: Adjusted prevalence, prevalence rank order, and relative risk for multimorbidity of the 46 chronic conditions in the multimorbid and non-multimorbid sample, ordered according to prevalence in total cohort

	Prevalence (%)		Prevalence rank		Risk ratio
	mm-sample	nmm-sample	mm-sample	nmm-sample	
Hypertension	65.4	17.9	1	1	3.7
Lipid metabolism disorders	42.9	5.6	2	3	7.7
Chronic low back pain	41.2	5.6	3	2	7.3
Severe vision reduction	21.5	4.8	7	5	4.5
Osteoarthrosis	29.5	3.4	4	7	8.8
Diabetes mellitus	28.5	4.8	5	4	6.0
Chronic ischemic heart disease	27.5	3.2	6	9	8.6
Thyroid diseases	21.4	3.3	8	8	6.5
Cardiac arrhythmias	17.6	1.9	10	11	9.3
Obesity	11.0	0.5	19	26	20.3
Purine/pyrimidine metabolism disorders/gout	16.1	1.0	12	20	16.2
Prostatic hyperplasia	10.9	1.9	20	12	5.8
Lower limb varicosis	18.7	1.6	9	14	12.1
Liver disease	10.4	0.6	21	25	18.1
Depression	13.7	1.5	14	15	9.2
Asthma/COPD	15.4	2.1	13	10	7.4
Noninflammatory gynecological problems	8.2	1.7	23	13	4.8
Atherosclerosis/PAOD	13.1	0.8	17	22	16.1
Osteoporosis	13.5	1.4	16	17	9.5
Renal insufficiency	5.5	0.2	31	42	25.5
Cerebral ischemia/chronic stroke	9.0	1.1	22	19	8.3
Cardiac insufficiency	12.8	1.0	18	21	13.0
Severe hearing loss	4.1	0.5	36	30	8.4

Chronic cholecystitis/gallstones	7.7	0.4	24	31	17.8
Somatoform disorders	5.8	0.4	29	33	13.9
Hemorrhoids	5.4	0.4	32	36	14.4
Intestinal diverticulosis	4.9	0.3	34	38	17.1
Rheumatoid arthritis/chronic polyarthritis	3.9	0.5	37	27	7.5
Cardiac valve disorders	4.9	0.4	35	35	12.8
Neuropathies	7.5	0.5	25	29	15.2
Dizziness	5.0	0.4	33	32	11.7
Dementia	5.6	1.4	30	16	3.9
Urinary incontinence	6.0	0.5	27	28	11.8
Urinary tract calculi	2.9	0.2	40	43	16.7
Anemia	3.6	0.2	38	40	15.8
Anxiety	2.4	0.2	41	44	14.4
Psoriasis	2.2	0.3	43	37	7.0
Migraine/chronic headache	3.1	0.3	39	39	12.3
Parkinson’s disease	2.3	0.4	42	34	5.9
Cancer	16.4	3.8	11	6	4.3
Allergy	6.0	0.6	28	23	9.7
Chronic gastritis/GERD	13.5	1.2	15	18	10.9
Sexual dysfunction	1.3	0.1	45	45	9.5
Insomnia	6.9	0.6	26	24	11.7
Tobacco abuse	1.1	0.1	46	46	14.9
Hypotension	2.0	0.2	44	41	8.9

mm-sample = multimorbid sample; nmm-sample = non-multimorbid sample
