# Supplementary material for: Which chronic diseases and disease combinations are specific to multimorbidity in the elderly? Results of a claims data based cross-sectional study in Germany
Source: BMC Public Health. 2011 Feb 14;11:101. doi: 10.1186/1471-2458-11-101 (PMC3050745; doi:10.1186/1471-2458-11-101)
Supplement: Additional file 4 — Adjusted prevalences, prevalence rank order and relative risk for multimorbidity of the 46 chronic conditions in the multimorbid and non-multimorbid sample in women according to prevalence in women sample. [file 1471-2458-11-101-S4.PDF]

Additional File 4: Adjusted prevalence, prevalence rank order, and relative risk for multimorbidity of the 46 chronic conditions in the multimorbid and non-multimorbid sample in women, ordered according to prevalence in women sample

	Prevalence (%)		Prevalence rank		Risk ratio
	mm-sample	nmm-sample	mm-sample	nmm-sample	
Hypertension	66.1	19.6	1	1	3.4
Chronic low back pain	42.9	6.1	2	2	7.0
Lipid metabolism disorders	42.3	6.1	3	3	7.0
Osteoarthrosis	33.1	4.1	4	7	8.2
Thyroid diseases	27.0	4.9	5	5	5.5
Diabetes mellitus	26.9	4.5	6	6	6.0
Severe vision reduction	22.4	5.2	9	4	4.4
Chronic ischemic heart disease	23.3	2.2	7	11	10.6
Lower limb varicosis	23.0	2.0	8	13	11.4
Osteoporosis	19.8	2.4	10	10	8.4
Depression	17.6	2.2	11	12	8.0
Cardiac arrhythmias	16.1	1.8	12	15	9.2
Cancer	13.6	3.1	15	9	4.4
Cardiac insufficiency	14.6	1.3	13	17	11.5
Asthma/COPD	14.2	1.7	14	16	8.3
Noninflammatory gynecological problems	13.3	3.1	17	8	4.3
Chronic gastritis/GERD	13.4	1.2	16	18	11.6
Purine/pyrimidine metabolism disorders/gout	12.0	0.4	18	32	31.4
Obesity	11.6	0.6	19	25	19.0
Atherosclerosis/PAOD	10.9	0.6	20	24	16.8
Chronic cholecystitis/gallstones	9.0	0.5	21	28	16.5
Liver disease	8.9	0.5	22	31	19.3
Cerebral ischemia/chronic stroke	8.1	1.0	23	19	7.9

Insomnia	7.8	0.7	24	22	11.2
Urinary incontinence	7.7	0.7	25	21	10.9
Dementia	6.8	2.0	27	14	3.4
Neuropathies	7.4	0.5	26	30	14.9
Somatoform disorders	6.7	0.6	28	27	11.6
Allergy	6.4	0.7	29	23	9.8
Dizziness	5.9	0.6	30	26	10.1
Intestinal diverticulosis	5.1	0.3	31	38	18.8
Rheumatoid arthritis/chronic polyarthritis	4.7	0.7	33	20	6.5
Cardiac valve disorders	4.7	0.3	34	35	14.6
Hemorrhoids	4.8	0.3	32	37	16.5
Migraine/chronic headache	4.0	0.4	36	33	11.3
Renal insufficiency	4.1	0.1	35	42	34.3
Severe hearing loss	3.7	0.5	37	29	7.3
Anemia	3.5	0.2	38	41	17.3
Anxiety	3.0	0.2	39	40	13.1
Parkinson`s disease	2.2	0.4	40	34	6.3
Hypotension	2.2	0.3	41	36	7.5
Psoriasis	1.9	0.2	42	39	8.4
Urinary tract calculi	1.7	0.1	43	43	27.0
Tobacco abuse	0.7	0.0	44	44	24.5
Sexual dysfunction	0.0	0.0	45	46	-
Prostatic hyperplasia	-	-	-	-	-

mm-sample = multimorbid sample; nmm-sample = non-multimorbid sample
