# Supplementary material for: Which chronic diseases and disease combinations are specific to multimorbidity in the elderly? Results of a claims data based cross-sectional study in Germany
Source: BMC Public Health. 2011 Feb 14;11:101. doi: 10.1186/1471-2458-11-101 (PMC3050745; doi:10.1186/1471-2458-11-101)
Supplement: Additional file 5 — Adjusted prevalences, prevalence rank order and relative risk for multimorbidity of the 46 chronic conditions in the multimorbid and non-multimorbid sample in men according to prevalence in the male sample. [file 1471-2458-11-101-S5.PDF]

Additional File 5: Adjusted prevalence, prevalence rank order, and relative risk for multimorbidity of the 46 chronic conditions in the multimorbid and non-multimorbid sample in men, ordered according to prevalence in the male sample

	Prevalence (%)		Prevalence rank		Risk ratio
	mm-sample	nmm-sample	mm-sample	nmm-sample	
Hypertension	64.3	15.8	1	1	4.1
Lipid metabolism disorders	43.9	5.0	2	4	8.8
Chronic low back pain	38.5	5.1	3	3	7.6
Chronic ischemic heart disease	34.3	4.5	4	6	7.7
Diabetes mellitus	31.2	5.1	5	2	6.1
Prostatic hyperplasia	28.3	4.2	6	8	6.7
Osteoarthrosis	23.7	2.5	7	10	9.6
Cancer	20.8	4.6	9	5	4.5
Purine/pyrimidine metabolism disorders/gout	22.7	1.8	8	12	12.9
Severe vision reduction	20.0	4.3	11	7	4.6
Cardiac arrhythmias	20.0	2.1	10	11	9.7
Asthma/COPD	17.3	2.6	12	9	6.8
Atherosclerosis/PAOD	16.6	1.0	13	16	16.2
Chronic gastritis/GERD	13.7	1.3	14	14	10.2
Thyroid diseases	12.5	1.4	16	13	8.9
Liver disease	13.0	0.7	15	19	17.9
Lower limb varicosis	11.8	1.0	17	17	12.1
Cerebral ischemia/chronic stroke	10.5	1.2	18	15	9.1
Obesity	10.2	0.5	19	26	22.1
Cardiac insufficiency	9.9	0.6	20	20	15.6
Neuropathies	7.7	0.5	22	23	15.6
Renal insufficiency	7.8	0.3	21	31	23.2
Depression	7.3	0.6	23	21	11.8

Hemorrhoids	6.4	0.5	24	24	13.3
Chronic cholecystitis/gallstones	5.7	0.3	25	35	19.1
Insomnia	5.4	0.5	26	27	12.0
Allergy	5.3	0.6	27	22	9.3
Cardiac valve disorders	5.1	0.4	28	28	11.4
Severe hearing loss	4.9	0.5	29	25	10.2
Urinary tract calculi	4.7	0.3	30	33	15.5
Intestinal diverticulosis	4.5	0.3	31	34	14.9
Somatoform disorders	4.3	0.2	32	41	20.1
Dementia	3.8	0.8	33	18	4.9
Anemia	3.6	0.3	34	39	14.5
Dizziness	3.5	0.2	35	40	15.4
Sexual dysfunction	3.4	0.3	37	32	10.9
Osteoporosis	3.4	0.3	36	37	13.0
Urinary incontinence	3.3	0.3	38	36	12.5
Psoriasis	2.7	0.4	39	29	6.4
Parkinson`s disease	2.4	0.4	41	30	5.6
Rheumatoid arthritis/chronic polyarthritis	2.4	0.3	40	38	9.5
Tobacco abuse	1.8	0.1	42	42	13.4
Migraine/chronic headache	1.6	0.1	43	44	13.2
Hypotension	1.6	0.1	44	43	12.1
Anxiety	1.3	0.1	45	45	16.0
Noninflammatory gynecological problems	-	-	-	-	-

mm-sample = multimorbid sample; nmm-sample = non-multimorbid sample
