# Supplementary material for: Which chronic diseases and disease combinations are specific to multimorbidity in the elderly? Results of a claims data based cross-sectional study in Germany
Source: BMC Public Health. 2011 Feb 14;11:101. doi: 10.1186/1471-2458-11-101 (PMC3050745; doi:10.1186/1471-2458-11-101)
Supplement: Additional file 6 — Triadic combinations with O/E ratio ≥ 1.5 out of the list of 100 most prevalent combinations in the multimorbid sample. [file 1471-2458-11-101-S6.PDF]

Additional File 6: Triadic combinations with O/E ratio ≥ 1.5 out of the list of 100 most prevalent combinations in the multimorbid sample

Preva-lence Rank	Combination of chronic conditions	O/E-ratio
63	Chronic low back pain + osteoarthrosis + osteoporosis	2.2
60	Lipid metabolism disorders + diabetes mellitus + purine/pyrimidine metabolism disorders/gout	1.9
55	Hypertension + diabetes mellitus + obesity	1.8
95	Chronic low back pain + osteoarthrosis + chronic gastritis/GERD	1.8
72	Lipid metabolism disorders + chronic ischemic heart disease + purine/pyrimidine metabolism disorders/gout	1.8
7	Hypertension + lipid metabolism disorders + purine/pyrimidine metabolism disorders/gout	1.6
26	Hypertension + diabetes mellitus + purine/pyrimidine metabolism disorders/gout	1.6
32	Lipid metabolism disorders + chronic low back pain + purine/pyrimidine metabolism disorders/gout	1.6
79	Lipid metabolism disorders + osteoarthrosis + purine/pyrimidine metabolism disorders/gout	1.6
92	Chronic low back pain + osteoarthrosis + purine/pyrimidine metabolism disorders/gout	1.6
61	Hypertension + chronic ischemic heart disease + atherosclerosis/PAOD	1.6
52	Chronic low back pain + osteoarthrosis + thyroid diseases	1.5
65	Lipid metabolism disorders + osteoarthrosis + lower limb varicosis	1.5
69	Hypertension + chronic ischemic heart disease + cardiac insufficiency	1.5
81	Hypertension + osteoarthrosis + obesity	1.5
86	Lipid metabolism disorders + chronic ischemic heart disease + cardiac arrhythmias	1.5

Prevalence rank: rank between 1 and 100 according to prevalence in the multimorbid sample; O/E ratio = observed-to-expected ratio.
