# Supplementary material for: Which chronic diseases and disease combinations are specific to multimorbidity in the elderly? Results of a claims data based cross-sectional study in Germany
Source: BMC Public Health. 2011 Feb 14;11:101. doi: 10.1186/1471-2458-11-101 (PMC3050745; doi:10.1186/1471-2458-11-101)
Supplement: Additional file 7 — Adjusted prevalence in total cohort and in mm-sample, and O/E-ratio of the 50 most prevalent triadic combinations of chronic conditions out of the list of 46. [file 1471-2458-11-101-S7.PDF]

Additional File 7: Adjusted prevalence in total cohort and in mm-sample, and O/E-ratio of the 50 most prevalent triadic combinations of chronic conditions from the list of 46

Rank	Combinations of chronic conditions	Prevalence in total cohort	Prevalence in mm-sample	O/E-ratio in mm-sample
1	Hypertension + lipid metabolism disorders + chronic low back pain (1, 2, 3)	7.5	12.0	1
2	Hypertension + chronic low back pain + osteoarthrosis (1, 3, 5)	6.4	10.4	1.3
3	Hypertension + lipid metabolism disorders + chronic ischemic heart disease (1, 2, 7)	5.8	9.4	1.2
4	Hypertension + lipid metabolism disorders + diabetes mellitus (1, 2, 6)	5.8	9.3	1.2
5	Hypertension + lipid metabolism disorders + osteoarthrosis (1, 2, 5)	5.4	8.6	1
6	Lipid metabolism disorders + chronic low back pain + osteoarthrosis (2, 3, 5)	4.7	7.5	1.4
7	Hypertension + lipid metabolism disorders + purine/pyrimidine metabolism disorders/gout (1, 2, 11)	4.6	7.3	1.6
8	Hypertension + chronic low back pain + chronic ischemic heart disease (1, 3, 7)	4.5	7.3	1
9	Hypertension + chronic low back pain + diabetes mellitus (1, 3, 6)	4.5	7.3	1
10	Hypertension + diabetes mellitus + chronic ischemic heart disease (1, 6, 7)	4.3	6.9	1.3
11	Hypertension + lipid metabolism disorders + thyroid diseases (1, 2, 8)	4.1	6.6	1.1
12	Hypertension + lipid metabolism disorders + severe vision reduction (1, 2, 4)	3.8	6.2	1
13	Hypertension + chronic low back pain + thyroid diseases (1, 3, 8)	3.8	6	1
14	Hypertension + chronic low back pain + lower limb varicosis (1, 3, 13)	3.7	6	1.2
15	Hypertension + chronic low back pain + Severe vision reduction (1, 3, 4)	3.6	5.8	1
16	Hypertension + osteoarthrosis + diabetes mellitus (1, 5, 6)	3.6	5.7	1
17	Hypertension + lipid metabolism disorders + lower limb varicosis (1, 2, 13)	3.5	5.7	1.1
18	Hypertension + osteoarthrosis + chronic ischemic heart disease (1, 5, 7)	3.4	5.5	1
19	Lipid metabolism disorders + chronic low back pain + chronic ischemic heart disease (2, 3, 7)	3.3	5.4	1.1
20	Hypertension + lipid metabolism disorders + cardiac arrhythmias (1, 2, 9)	3.3	5.3	1.1
21	Hypertension + severe vision reduction + diabetes mellitus (1, 4, 6)	3.2	5.1	1.3
22	Hypertension + osteoarthrosis + lower limb varicosis (1, 5, 13)	3.1	5.1	1.4
23	Hypertension + chronic low back pain + purine/pyrimidine metabolism disorders/gout (1, 3, 11)	3.1	5.1	1.2
24	Lipid metabolism disorders + chronic low back pain + diabetes mellitus (2, 3, 6)	3	4.8	1
25	Hypertension + chronic low back pain + cardiac arrhythmias (1, 3, 9)	3	4.8	1

26	Hypertension + diabetes mellitus + purine/pyrimidine metabolism disorders/gout (1, 6, 11)	2.9	4.7	1.6
27	Hypertension + lipid metabolism disorders + atherosclerosis/PAOD (1, 2, 18)	2.9	4.7	1.3
28	Chronic low back pain + osteoarthrosis + Lower limb varicosis (3, 5, 13)	2.9	4.6	2
29	Lipid metabolism disorders + chronic low back pain + thyroid diseases (2, 3, 8)	2.8	4.5	1.2
30	Lipid metabolism disorders + chronic low back pain + lower limb varicosis (2, 3, 13)	2.8	4.5	1.4
31	Hypertension + chronic ischemic heart disease + cardiac arrhythmias (1, 7, 9)	2.8	4.5	1.4
32	Lipid metabolism disorders + chronic low back pain + purine/pyrimidine metabolism disorders/gout (2, 3, 11)	2.8	4.5	1.6
33	Lipid metabolism disorders + diabetes mellitus + chronic ischemic heart disease (2, 6, 7)	2.7	4.4	1.3
34	Hypertension + chronic low back pain + osteoporosis (1, 3, 19)	2.7	4.4	1.2
35	Hypertension + osteoarthrosis + thyroid diseases (1, 5, 8)	2.7	4.4	1.1
36	Chronic low back pain + osteoarthrosis + chronic ischemic heart disease (3, 5, 7)	2.7	4.4	1.3
37	Hypertension + chronic low back pain + chronic gastritis/GERD (1, 3, 42)	2.7	4.3	1.2
38	Hypertension + lipid metabolism disorders + obesity (1, 2, 10)	2.7	4.3	1.4
39	Hypertension + chronic low back pain + asthma/COPD (1, 3, 16)	2.7	4.3	1
40	Hypertension + diabetes mellitus + thyroid diseases (1, 6, 8)	2.6	4.2	1.1
41	Hypertension + Severe vision reduction + osteoarthrosis (1, 4, 5)	2.6	4.2	1
42	Hypertension + lipid metabolism disorders + cancer (1, 2, 40)	2.6	4.2	0.9
43	Hypertension + chronic low back pain + depression (1, 3, 15)	2.6	4.2	1.1
44	Hypertension + severe vision reduction + chronic ischemic heart disease (1, 4, 7)	2.6	4.1	1.1
45	Hypertension + lipid metabolism disorders + asthma/COPD (1, 2, 16)	2.6	4.1	1
46	Chronic low back pain + osteoarthrosis + diabetes mellitus (3, 5, 6)	2.6	4.1	1.2
47	Lipid metabolism disorders + chronic low back pain + severe vision reduction (2, 3, 4)	2.5	4.1	1.1
48	Hypertension + lipid metabolism disorders + liver disease (1, 2, 14)	2.5	4.1	1.4
49	Hypertension + lipid metabolism disorders + chronic gastritis/GERD (1, 2, 42)	2.5	4	1
50	Hypertension + chronic ischemic heart disease + purine/pyrimidine metabolism disorders/gout (1, 7, 11)	2.5	4	1.4

mm-sample = multimorbid sample; O/E ratio = observed-to-expected ratio; brackets = ICD codes (see Additional File 2)
